# 6 Risk Factors and Comorbidities Associated with Post-burn Hypertension

**DOI:** 10.1093/jbcr/irac012.010

**Published:** 2022-03-23

**Authors:** Tsola A Efejuku, Alejandro A Joglar, Shivan N Chokshi, Kassandra K Corona, Kendall Wermine, Sunny Gotewal, Giovanna De La Tejera, George Golovko, Maria Haseem, Phillip H Keys, Juquan Song, Steven E Wolf, Amina El Ayadi

**Affiliations:** University of Texas Medical Branch, Galveston, Texas; University of Texas Medical Branch, Galveston, Texas; University of Texas Medical Branch at Galveston, Galveston, Texas; University of Texas Medical Branch, Galveston, Texas; University of Texas Medical Branch, Galveston, Texas; University of Texas Medical Branch, Galveston, Texas; University of Texas Medical Branch, Galveston, Texas; University of Texas Medical Branch at Galveston, Galveston, Texas; University of Texas Medical Branch, Galveston, Texas; University of Texas Medical Branch, Galveston, Texas; University of Texas Medical Branch, Galveston, Texas; University of Texas Medical Branch at Galveston, Galveston, Texas; University of Texas Medical Branch at Galveston, Galveston, Texas

## Abstract

**Introduction:**

Hypertension (HTN) is a prevalent condition in the United States and leads to an increased risk of developing other comorbidities. However, the impact of hypertension following severe burns on patient outcomes is not known. We hypothesize that post-burn hypertension is associated with an increased risk of other comorbidities and mortality.

**Methods:**

This study used data from TriNetX, a global federated health research network. Burned patients who were diagnosed with essential hypertension at least 1 day after injury were identified in the TriNetX database using specific ICD codes and were compared to those who did not develop essential hypertension; neither cohort was diagnosed with hypertension prior to injury. Each cohort was balanced for age, gender, race, and ethnicity. Occurrence of the following within 3 days of burn was compared between the two cohorts: acute kidney injury (AKI), hyperglycemia, heart failure, coronary artery disease, and death. These patient cohorts were then stratified by gender, percent total body surface area (TBSA) burned, and age. Statistical analysis for the measures of association used an odds ratio with a 95% confidence interval and a risk ratio with a z-test. Significance for the z-test was set at a p-value of < 0.05.

**Results:**

The search identified 460,977 burn patients of whom 87,808 were diagnosed with hypertension at least 1 day after burn injury. Those diagnosed with hypertension were 7.25 times as likely to develop AKI, 5.45 times as likely to develop hyperglycemia, 7 times as likely to develop heart failure, 7.17 times as likely to develop coronary artery disease, and 1.78 times as likely to die. Men were at greater risk of experiencing AKI, heart failure, coronary artery disease, and death, however, women were 1.51 times as likely to develop hyperglycemia. Stratification based on % TBSA burned indicated an increased risk for all outcomes for patients with a high percentage of total body surface area burned (60% to > 90% TBSA burned was higher than < 10% to 50-59% groups). Subgroup analysis based on age indicated elevated risk of developing AKI, heart failure, coronary artery disease, or death with age. However, we found a spike in risk for all outcomes in the 0-9 age group. All data was significant at p < .0001.

**Conclusions:**

A new hypertension diagnosis in severely burned patients is highly associated with other comorbidities including acute kidney injury, heart failure, coronary artery disease, and death. Overall, males, older patients, and those with a higher percent TBSA burned are at a higher risk of developing these comorbidities.

